# Topics of Public Interest

**Published:** 1916-02

**Authors:** 


### Topics of Public Interest.

**Fatal Second Attack of Typhoid.** Our attention was called to this case by a press report and we wrote to Dr. Ellis, Health Officer of Dunkirk for verification. Dr. Walter H. Vosburg of Dunkirk has replied, giving the following details: The patient had typhoid at the age of five. The history on this point is apparently reliable. Three years ago, an attack of acute articular rheumatism left the patient with a pronounced mitral regurgitation and, a year ago, he was run over by an auto. Last month he developed indubitable typhoid, at first as an ambulant affection. Rose spots appeared on the sixth day and the Widal reaction was positive the same day. The patient died, the attack of typhoid being severe and the patient handicapped by the previous cardiac lesion and the injury. Age at death, 13 years. This case was, at the time, the only one of typhoid in Dunkirk.

